# Alpha9alpha10 knockout mice show altered physiological and behavioral responses to signals in masking noise

**DOI:** 10.1121/10.0025985

**Published:** 2024-05-13

**Authors:** Jane A. Mondul, Kali Burke, Barbara Morley, Amanda M. Lauer

**Affiliations:** 1 Department of Otolaryngology–Head and Neck Surgery, Johns Hopkins University School of Medicine, Baltimore, Maryland 21205, USA; 2 Boys Town National Research Hospital, Omaha, Nebraska 68131, USA; 3 Department of Neuroscience, Johns Hopkins University School of Medicine, Baltimore, Maryland 21205, USA

## Abstract

Medial olivocochlear (MOC) efferents modulate outer hair cell motility through specialized nicotinic acetylcholine receptors to support encoding of signals in noise. Transgenic mice lacking the alpha9 subunits of these receptors (α9KOs) have normal hearing in quiet and noise, but lack classic cochlear suppression effects and show abnormal temporal, spectral, and spatial processing. Mice deficient for both the alpha9 and alpha10 receptor subunits (α9α10KOs) may exhibit more severe MOC-related phenotypes. Like α9KOs, α9α10KOs have normal auditory brainstem response (ABR) thresholds and weak MOC reflexes. Here, we further characterized auditory function in α9α10KO mice. Wild-type (WT) and α9α10KO mice had similar ABR thresholds and acoustic startle response amplitudes in quiet and noise, and similar frequency and intensity difference sensitivity. α9α10KO mice had larger ABR Wave I amplitudes than WTs in quiet and noise. Other ABR metrics of hearing-in-noise function yielded conflicting findings regarding α9α10KO susceptibility to masking effects. α9α10KO mice also had larger startle amplitudes in tone backgrounds than WTs. Overall, α9α10KO mice had grossly normal auditory function in quiet and noise, although their larger ABR amplitudes and hyperreactive startles suggest some auditory processing abnormalities. These findings contribute to the growing literature showing mixed effects of MOC dysfunction on hearing.

## I. INTRODUCTION

The medial olivocochlear (MOC) efferent system modulates afferent encoding of incoming sounds primarily via cholinergic inhibition of the outer hair cells (Fuchs and Lauer, 2019). MOC neurons reduce outer hair cell motility through specialized nicotinic acetylcholine receptors (nAChRs) comprised of alpha9 and alpha10 subunits (Elgoyhen *et al.*, 1994; Elgoyhen *et al*., 2001), thus serving as a gain control for the cochlear amplifier. Previous work using lesions or stimulations of the olivocochlear bundle have indicated a role in encoding of signals in noise and protection from acoustic injury (Boero *et al.*, 2018; Elgoyhen, 2020; Guinan, 2006; Kawase and Liberman, 1993; Liberman and Guinan, 1998; Lopez-Poveda, 2018; Maison *et al.*, 2013). However, demonstrating clear and consistent behavioral effects of olivocochlear manipulations has been less straightforward (Lauer *et al.*, 2022).

Much of what is known about MOC function has been derived from studies in alpha9 knockout (α9KO) transgenic mice (Vetter *et al.*, 1999). These mice were generated to have a null mutation in the alpha9 subunit of nAChRs that results in the loss of the classic inhibitory effects on cochlear activity as measured via suppression of compound action potentials and otoacoustic emissions by electrical stimulation. Binding of acetylcholine to the outer hair cell nAChRs causes calcium-mediated potassium influx, which hyperpolarizes the cell (Fuchs, 2014). α9KO mice show structural changes to MOC innervation patterns, including fewer but larger MOC terminals per outer hair cell and disrupted patterns of tunneling fibers (Boero *et al.*, 2018; Lauer and May, 2011; Morley *et al.*, 2017; Vetter *et al.*, 1999), suggesting that functional alpha9 nAChR subunits are necessary for normal development and function of the MOC system.

The hearing abilities of α9KOs have been probed using a variety of physiological and behavioral methods in order to better understand the role of the MOC system in hearing. α9KO mice have normal hearing sensitivity for tones in quiet as measured by auditory brainstem responses (ABRs) (Lauer, 2017; Lauer and May, 2011; May *et al.*, 2002; Morley *et al.*, 2017; Vetter *et al.*, 1999), distortion product otoacoustic emissions (Lauer and May, 2011; Morley *et al.*, 2017), and behavior (May *et al.*, 2002; Prosen *et al.*, 2000). Surprisingly, α9KOs also show normal tone detection, intensity discrimination, and prepulse inhibition (PPI) in noise (Allen and Luebke, 2017; May *et al.*, 2002). However, α9KOs show weak MOC reflexes (Chambers *et al.*, 2012; Vetter *et al.*, 1999; but see Morley *et al.*, 2017), abnormal temporal processing (Lauer and May, 2011), reduced PPI in quiet (Allen and Luebke, 2017), and impaired frequency resolution and spatial hearing (Clause *et al.*, 2017). Overall, α9KO mouse studies seem to refute the hypothesis that the MOC system plays a role in hearing-in-noise or suggest that other compensatory mechanisms take over when MOC activity is disrupted. The deficits observed in other suprathreshold processing abilities are likely related to abnormal development of the central auditory system (Clause *et al.*, 2014).

Another transgenic model that is deficient for both the alpha9 and alpha10 subunits of nAChRs was generated by Morley *et al.* (2017). The alpha9alpha10 double KO model (α9α10KO) was developed to investigate whether the deletion of both subunits would produce a more severe MOC lesion phenotype than the α9KO. Morley *et al.* (2017) found that α9α10KOs have comparable ABR thresholds and distortion product otoacoustic emission amplitudes to both wild-typed (WTs) and α9KOs. However, α9α10KOs have weaker MOC reflexes than WTs, α9KOs, and α10KOs. Further characterization of hearing abilities in the α9α10KO mouse model could provide additional evidence toward hypothesized roles of the MOC system. In the present study, we aimed to characterize auditory function more comprehensively in adult α9α10KO mice by using both physiological and reflex-based behavioral methods to probe hearing-in-noise and suprathreshold processing abilities in untrained animals. Specifically, we measured: 1) ABR thresholds, amplitudes, and latencies to sounds in quiet and in noise; 2) acoustic startle responses (ASRs) to sounds in quiet and in noise; and 3) frequency and intensity difference (ID) sensitivity using PPI of the ASR. Data will be discussed in the context of prior studies in other MOC mutant mice that used similar physiological and behavioral assays.

## II. METHODS

### A. Subjects

Experiments were conducted in 3-month-old mice of two genotypes: C57BL/6J (WT controls; *n* = 7, 3 female) and alpha9alpha10 double KOs (α9α10KO; *n* = 9, 4 female). Mice were obtained from the Morley laboratory at Boys Town National Research Hospital. WT controls were from the same colony as the α9α10KO mice and obtained by mating heterozygote females with heterozygote or WT males or WT females with heterozygote males. Littermate controls were not possible due to the breeding strategy employed. Background strain, generation, and validation of the transgenic line were previously described (Morley *et al.*, 2017). Briefly, the alpha9 and alpha10 KO mice were backcrossed to >99% congenicity on the C57BL/6J background using MAXBAX (Charles River, Troy, NY). The alpha9 and alpha10 KO mice were crossed to construct the double KO. The double KO genotype was uncrossed by breeding with WT C57BL/6J mice obtained from Jackson Labs (Bar Harbor, ME). KOs were uncrossed and re-crossed yearly. This results in a background in the double KO mouse with greater similarity than would be found with a simple cross-of the alpha9 and alpha10 genotypes. Since the WT and α9α10KO mice were both on a C57BL/6J background, all animals possessed the mutant version of *Cdh23.*

Prior to testing, animals were group housed in high-traffic mouse vivaria until 2 months of age, transported to Johns Hopkins University, and acclimated to the new housing facility for 4 weeks. Mice were exposed to unknown levels of noise during transport, but this was a common factor for all animals tested in this study. At the time of testing, animals were group-housed in a low noise vivarium; sound levels were previously described (Wu *et al.*, 2020). The housing room was maintained on a 12:12 h light:dark cycle (7:00 to 19:00). Up to five mice were housed per one filter top shoebox cage (30 × 19 × 13 cm^3^) with corncob bedding and nestlets. Exclusion criteria included abnormal hearing thresholds in quiet or signs of outer or middle ear infections; however, no animals were excluded from any of these experiments. Mice weighed between 20 and 30 g at the time of testing, with no significant difference across genotypes. All procedures were approved by the Johns Hopkins University Animal Care and Use Committee and follow the National Institutes of Health ARRIVE Guidelines.

### B. Procedures

#### 1. ABRs in quiet and in masking noise

ABR testing procedures were similar to those previously described in this laboratory (e.g., Capshaw *et al.*, 2022; Vicencio-Jimenez *et al.*, 2021). Briefly, ABR testing was conducted in a sound-treated booth (Industrial Acoustics Company, Bronx, NY; 59 × 74 × 60 cm^3^) lined with acoustic foam (Pinta Acoustic, Minneapolis, MN). Mice were anesthetized with an intraperitoneal injection of 100 mg/kg ketamine and 20 mg/kg xylazine and placed on a heating pad to maintain a temperature of 37 °C. Subdermal needle electrodes (Disposable Horizon, 13 mm needle, Rochester Med, Coral Springs, FL) were placed on the vertex (active), ipsilateral mastoid (reference), and hind limb (ground) in a standard ABR recording montage. One ear was tested per subject (random and counterbalanced selection). ABR signals were acquired with a Medusa4Z preamplifier (12 kHz sampling rate) and filtered from 300–3000 Hz with an additional band-reject filter at 60 Hz. *Post hoc* filters from 300–3000 Hz with steeper cutoff slopes were also applied for additional smoothing.

ABRs in quiet were recorded to clicks (100 *μ*s) and tone bursts (4, 8, 12, 16, 24, 32 kHz; 5 ms duration, 0.5 ms rise/fall) at a rate of 21/s for a total of 512 presentations with alternating stimulus polarities. Stimuli were created in SigGen software (Tucker-Davis Technologies [TDT], Alachua, FL) and generated by a RZ6 multi-I/O processor (TDT). Stimuli were played from a free field speaker (MF1, TDT) located 10 cm from the animal's ear canal at 0° azimuth. Stimuli were calibrated with a 0.25 in. free-field microphone (PCB Piezotronics, Depew, NY, model 378C01) placed at the location of the animal's ear canal. Stimulus level ranged from 90 to 10 dB SPL in 10 dB steps.

Masked ABRs were recorded to clicks and tone bursts (4, 8, 12, 16 kHz) in the presence of a 50 dB SPL broadband noise (4–20 kHz; 8 dB SPL spectrum level). Masking noise was generated by an Elgenco 602 A gaussian noise generator and presented from a separate free field speaker (MF1, TDT) located adjacent to the stimulus speaker and within the minimum audible angle of mice (Behrens and Klump, 2016; Heffner *et al.*, 2001; Lauer *et al.*, 2011). The sound level was calibrated with a Larson Davis sound level meter (model 824) with Z-weighting prior to each recording session. The testing order of ABRs in quiet and in noise was randomized across animals.

ABR traces were analyzed offline by two researchers, one of whom was blinded to the subject and stimulus condition. Inter-rater reliability was >0.85 for ABR thresholds, amplitudes, and latencies. ABR threshold was defined as the average between the lowest sound level to evoke a response and the first level with no response (any wave). Peak-to-trough amplitudes and peak latencies were derived for ABR Waves I, II III, and IV using manual peak-picking methods and a semi-automated ABR wave analysis software previously described (Burke *et al.*, 2023). Due to variability in central wave morphologies, amplitude and latency analyses were focused on Wave I only. ABR threshold differences between noise and quiet (dB masking) and ratios between ABR Wave I amplitudes in quiet and in noise were calculated to probe the effects of masking noise.

#### 2. ASR and PPI general procedures

ASR and PPI testing procedures were similar to those previously described (e.g., Clause *et al.*, 2017; Kim *et al.*, 2022). Briefly, mice were brought into the testing room to acclimate 30 min prior to testing. Animals were tested one at a time, and the order of tasks (ASR in quiet, ASR in noise, PPI frequency, PPI intensity) was pseudorandomized. All startle experiments were conducted by the same experimenter during the daytime light cycle of the animals' housing, between 8:00 and 18:00.

Testing was conducted in a sound-treated booth (Industrial Acoustics Company; 37 × 53 × 33 cm^3^) lined with acoustic foam (Pinta Acoustic, Minneapolis, MN). Stimuli and masking noise were delivered from two adjacent speakers (RadioShack Super Tweeter) located on one end of the sound booth. Stimuli were generated by an RP2.1 real time processor (TDT), a PA5 programmable sound attenuator (TDT), and an amplifier (Crown D75A). Speakers were located 15 cm from the animal's head, and intensity was calibrated using a Larson Davis Sound Level Meter (model 824) with z-weighting.

During testing, mice were placed into an acoustically transparent mouse restraint device (7.2 × 3.3 × 2.8 cm^3^) atop a piezoelectric accelerometer in the center of the sound-treated booth. Movements of the animal were recorded as voltage. For all tests, a random intertrial waiting period of 5–15 s was used to prevent subjects from predicting the onset of a trial, followed by a 5 s quiet period with a noise criterion of <0.4 V to ensure the animal was still prior to the onset of the stimulus. The subject's startle response was recorded over 120 ms following the onset of the startle eliciting stimulus. ASR amplitude was defined as the maximum peak-to-peak voltage during the 120 ms recording window. Testing was conducted over two sessions per animal on separate days, with each session lasting 35–45 min each, in order to avoid habituation of the startle response. Animals were returned to their home cage after testing. Data were screened offline to ensure that all trials counted as startles had appropriate latency and amplitude values.

#### 3. ASR in quiet and in masking noise

ASRs were measured in response to noise bursts (20 ms) in quiet and in the presence of continuous 60 dB SPL broadband noise (4–20 kHz). Pulses were pseudorandomly presented at levels of 70, 80, 90, 100, and 105 dB SPL for a minimum of 10 times per level.

#### 4. ASR frequency and ID sensitivity

ASR tasks probing frequency and ID sensitivity were conducted in the presence of a 65 dB SPL 10 kHz tone background. Startle eliciting stimuli for both tasks were 20 ms broadband noise burst presented at 105 dB SPL. The startle noise bursts were presented immediately after a prepulse cue of altered tone frequency or tone intensity, which had a duration of 80 ms. For the frequency difference (FD) task, the background tone frequency changed from 10 kHz to one of eight off frequencies (7, 8, 9, 9.5, 10.5, 11, 12, 13 kHz) in a pseudorandom order. For the ID task, the background tone level changed from 70 dB SPL to one of five other intensities (72, 74, 76, 78, 80) in a pseudorandom order. Prepulse cue conditions were pseudorandomly presented for a minimum of 10 times per cue, including no change trials. Since *d′*-like estimates of sensitivity do not exceed 1.0 in PPI tests, we did not calculate frequency or ID limens or thresholds for this experiment (Kim *et al.*, 2022; Lauer *et al.*, 2017).

### C. Statistical analyses

To determine whether α9α10KO mice had different hearing sensitivity in quiet or noise conditions, we used linear mixed-effects models (lmer in the lme4 R package, RRID: SCR_015654) to assess the effects of genotype, sex, and stimulus frequency or level on dependent variables, such as ABR thresholds, ABR Wave I amplitudes and amplitude ratios, ASR magnitude, and PPI. Genotype, sex, stimulus frequency, and stimulus level were treated as categorical factors. Although sex was included in the models, group sizes were small, so sex differences have been deemphasized in this manuscript and should be interpreted with caution. We controlled for individual dependencies in our data by including a random intercept for mouse identity. Model selection was done using the step-up method for linear mixed effects modeling and the goodness of fit for our model was measured using the Akaike information criterion. We present the results for the ANOVA based on each model, as well as *post hoc* tests controlling for multiple comparisons using the mvt adjustment (emmeans R package, RRID: SCR_018734). Tukey's *post hoc* analyses were performed to assess significance (emmeans R package, RRID: SCR_018734).

## III. RESULTS

### A. ABRs in quiet and in noise

**FIG. 1.**
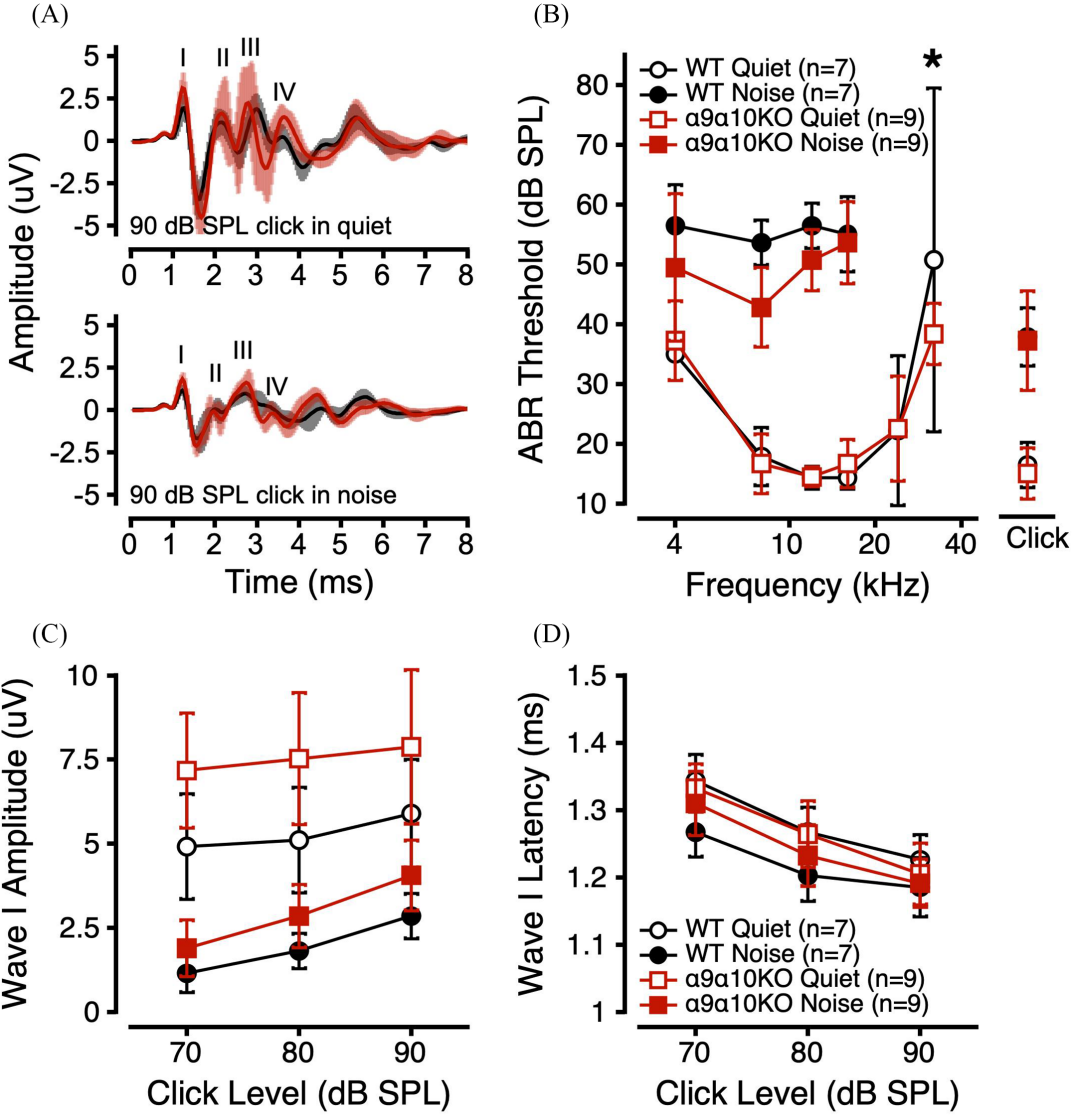
(Color online) ABRs in quiet and noise for α9α10KO and WT mice. (A) Mean (±1 standard deviation). ABR waveforms in response to a 90 dB SPL click in quiet (top) and noise (bottom) for α9α10KO (trace in foreground) and WT mice (trace in background). ABR thresholds as a function of stimulus frequency (B), Wave I amplitudes to suprathreshold clicks (C), and Wave I latencies to suprathreshold clicks (D) in quiet (open symbols) and noise (filled symbols) for α9α10KO (squares) and WT mice (circles). Error bars depict ±1 standard deviation from the mean. *indicates a statistically significant difference across genotypes (p-value <0.05).

ABRs were measured to clicks and tone bursts in quiet and in 50 dB SPL masking noise. Mean waveforms are shown for each genotype in response to a 90 dB SPL click in quiet and in noise [Fig. 1(A), top and bottom panels]. Wave morphology was generally as expected for α9α10KO mice, with all waves present in quiet and in noise but a less robust waveform in noise. α9α10KO mouse ABR waveforms appeared more variable across individual animals than for the WT mice, especially in quiet and for later wave components. For both genotypes, the addition of masking noise caused an elevation of ABR thresholds [Fig. 1(B)] and reduction of ABR amplitudes [Fig. 1(C)], but had minimal effects on Wave I latency [Fig. 1(D)].

Overall, ABR thresholds in quiet and in noise were not different between WT and α9α10KO mice, except for higher and more variable thresholds at 32 kHz in the WTs [Fig. 1(B)]. Linear mixed-effects models comparing ABR thresholds in quiet across genotype, stimulus (click or tone burst frequency), and sex revealed significant main effects of stimulus and sex, with significant interactions between sex/stimulus and genotype/stimulus (Table I). *Post hoc* analyses revealed a significant threshold difference at 32 kHz across genotypes (*p* = 0.0033). Linear mixed-effects models comparing ABR thresholds in noise across genotype, stimulus, and sex revealed significant main effects of genotype and stimulus, with a significant three-way interaction between genotype, sex, and stimulus (Table I). *Post hoc* analyses revealed a significant difference between male WT and α9α10KO ABR thresholds in noise at 4 and 8 kHz (p = 0.0095 and 0.0038, respectively).

**TABLE I.** Results from linear mixed effects model statistics on all measures.

Test	Variables	F	Df	p	*η* ^2^
	Genotype	0.6849	1, 16.172	0.419 940	0.002 0
	Stimulus	47.3743	6, 88.297	**<2.2 × 10^–16^** ^a^	0.821 6
	Sex	5.9380	1, 16.172	**0.026 733** ^a^	0.017 2
ABR threshold - quiet	Genotype:Stimulus	2.4807	6, 88.297	**0.028 972** ^a^	0.043 0
	Genotype:Sex	3.4664	1, 16.172	0.080 903	0.010 0
	Stimulus:Sex	3.9620	6, 88.297	**0.001 482** ^a^	0.068 7
	Genotype:Stimulus:Sex	2.1636	6, 88.297	0.053 990	0.037 5
	Genotype	11.9893	1, 77	**0.000 876 7** ^a^	0.100 5
	Stimulus	21.6681	4, 77	**5.272 × 10^–12^** ^a^	0.726 7
	Sex	0.0037	1, 77	0.951 456 7	0.000 0
ABR threshold - noise	Genotype:Stimulus	1.4976	4, 77	0.211 203 5	0.050 2
	Genotype:Sex	0.5793	1, 77	0.448 919 1	0.004 8
	Stimulus:Sex	0.9115	4, 77	0.461 607	0.030 6
	Genotype:Stimulus:Sex	2.5995	4, 77	**0.042 535 5** ^a^	0.087 2
	Genotype	9.7382	1, 16.704	**0.006 326** ^a^	0.064 45
	Stimulus	28.0181	4, 59.718	**4.067 × 10^–13^** ^a^	0.741 65
dB masking	Sex	0.6149	1, 16.704	0.443 922	0.004 06
ABR threshold (dB SPL)	Genotype:Stimulus	1.3051	4, 59.718	0.278 445	0.034 54
Noise – quiet	Genotype:Sex	0.8515	1, 16.704	0.369 279	0.005 64
	Stimulus:Sex	1.5878	4, 59.718	0.189 294	0.042 03
	Genotype:Stimulus:Sex	4.0641	4, 59.718	**0.005 570** ^a^	0.107 60
	Genotype	9.3518	1, 16	**0.007 510 8** ^a^	0.061 30
	dB	48.2807	2, 32	**2.171 × 10^–10^** ^a^	0.632 96
	Sex	2.4999	1, 16	0.133 417 3	0.016 38
Click Wave 1 amplitude	Genotype:dB	4.7463	2, 32	**0.015 662 4** ^a^	0.062 22
Quiet	Genotype:Sex	7.8429	1, 16	**0.012 827 5** ^a^	0.051 41
	dB:Sex	1.2114	2, 32	0.311 065 1	0.015 88
	Genotype:dB:Sex	12.1910	2, 32	**0.000 115 9** ^a^	0.159 82
	Genotype	0.8449	1, 16	0.371 64	0.001 05
	dB	393.575	2, 32	**<2.2 × 10^–16^** ^a^	0.977 22
	Sex	1.9181	1, 16	0.185 08	0.002 37
Click Wave 1 latency	Genotype:dB	2.3564	2, 32	0.111 00	0.005 84
Quiet	Genotype:Sex	3.0129	1, 16	0.101 82	0.003 74
	dB:Sex	0.3927	2, 32	0.678 44	0.000 97
	Genotype:dB:Sex	3.5340	2, 32	**0.041 04** ^a^	0.008 77
	Genotype	7.8229	1, 16	**0.012 922 6** ^a^	0.009 37
	dB	397.233	2, 32	**<2.2 × 10^–16^** ^a^	0.951 84
	Sex	0.5956	1, 16	0.451 515 6	0.000 71
Click Wave 1 amplitude	Genotype:dB	4.2058	2, 32	**0.023 894 2** ^a^	0.010 07
Noise	Genotype:Sex	3.6257	1, 16	0.075 035 6	0.004 34
	dB:Sex	0.4160	2, 32	0.663 205 9	0.000 99
	Genotype:dB:Sex	9.4520	2, 32	**0.000 594 8** ^a^	0.022 64
	Genotype	2.1698	1, 16	0.160 147	0.007 12
	dB	137.031	2, 32	**<2.2 × 10^–16^** ^a^	0.900 17
	Sex	0.0334	1, 16	0.857 358	0.000 11
Click Wave 1 latency	Genotype:dB	4.5833	2, 32	**0.017 77** ^a^	0.030 10
Noise	Genotype:Sex	14.1853	1, 16	**0.001 689** ^a^	0.046 59
	dB:Sex	2.3049	2, 32	0.116 098	0.015 14
	Genotype:dB:Sex	0.1138	2, 32	0.892 800	0.000 74
Wave 1 N/Q ratio	Genotype	0.6732	1, 16	0.424	0.001 96
Click	dB	171.861	2, 32	**<2.2 × 10^–16^** ^a^	0.998 04
Wave 1 N/Q ratio – 4 kHz	NS from intercept				
	Genotype	10.4473	1, 15	**0.005 583 0** ^a^	0.203 49
	dB	10.1291	2, 30	**0.000 435 2** ^a^	0.394 58
	Sex	5.7751	1, 15	**0.029 641 8** ^a^	0.112 48
Wave 1 N/Q ratio – 8 kHz	Genotype:dB	4.1557	2, 30	**0.025 520 4** ^a^	0.161 88
	Genotype:Sex	0.5069	1, 15	0.487 411 7	0.009 87
	dB:Sex	0.0474	2, 30	0.953 822 7	0.001 84
	Genotype:dB:Sex	2.9734	2, 30	0.066 362 6	0.115 83
Wave 1 N/Q ratio – 12 kHz	NS from intercept				
	Genotype	0.5333	1, 12	0.479 25	0.008 07
	dB	28.1579	2, 24	**5.069 × 10^–7^** ^a^	0.852 06
Wave 1 N/Q ratio – 16 kHz	Sex	1.278	1, 12	0.280 36	0.019 33
	Genotype:dB	3.9831	2, 24	**0.032 08** ^a^	0.120 52
ASR - quiet	dB	59.693	4, 784	**<2.2 × 10^–16^** ^a^	1
ASR - noise	dB	74.099	4, 784	**<2.2 × 10^–16^** ^a^	1
	Genotype	5.1783	1, 16.01	**0.036 96** ^a^	0.103 28
ASR FD raw	Frequency	4.2441	8, 1336.02	**4.793 × 10^–5^** ^a^	0.677 23
	Sex	3.3860	1, 16.01	0.084 37	0.067 53
	Genotype:Frequency	0.9521	8, 1336.02	0.472 21	0.151 93
	Genotype	0.4816	1, 16	0.497 6	0.007 5
ASR FD PPI	Frequency	6.1751	8, 128	**9.905 × 10^–7^** ^a^	0.768 9
	Sex	0.0035	1, 16	0.953 9	0.000 1
	Genotype:Frequency	1.7948	8, 128	0.083 8	0.223 5
	Genotype	6.8083	1, 16.04	**0.018 95** ^a^	0.214 48
ASR ID raw	dB	3.0099	5, 1231.06	**0.010 46** ^a^	0.474 10
	Sex	5.2541	1, 16.04	**0.035 75** ^a^	0.165 52
	Genotype:dB	0.9261	5, 1231.06	0.462 99	0.145 88
ASR ID PPI	dB	3.1389	5, 80	**0.012 25** ^a^	1

^a^ Boldface numbers indicate statistically significant p-values (<0.05).

To evaluate the effects of masking noise on α9α10KO ABR thresholds, we calculated the amount of masking threshold shift for each subject and stimulus (*dB Masking* = *ABR Threshold_Noise_* *–* *ABR Threshold_Quiet_*). Since the addition of masking noise elevates ABR thresholds, dB masking values across genotypes and stimuli were greater than 0 dB SPL. α9α10KO mice exhibited less masking (i.e., smaller dB masking values) than WTs (Table II). Linear mixed-effects models comparing dB masking values across genotype, stimulus, and sex revealed significant main effects of genotype and stimulus, with a significant three-way interaction of genotype, stimulus, and sex (Table I). *Post hoc* analyses indicated significant genotype differences in dB masking values for males at 4 kHz (p = 0.0038) and 8 kHz (p = 0.0050) and for females at 16 kHz (p = 0.0187). Overall, these results suggest that α9α10KO mouse ABRs were less susceptible to masking noise than WTs.

**TABLE II.** Descriptive statistics of ABR masking (dB SPL) by genotype, sex, and stimulus.

Genotype	Sex	*n*	Stimulus	Mean	SD
WT	F	3	4	20	0
WT	F	3	8	30	0
WT	F	3	12	41.66667	2.886 751
WT	F	3	16	45	7.071 068
WT	F	3	Click	16.66667	5.773 503
WT	M	4	4	22.5	9.574 271
WT	M	4	8	40	0
WT	M	4	12	42.5	5
WT	M	4	16	38.75	6.291 529
WT	M	4	Click	25	5.773 503
α9α10KO	F	4	4	20	14.142 14
α9α10KO	F	4	8	28.75	6.291 529
α9α10KO	F	4	12	37.5	5
α9α10KO	F	4	16	25	7.071 068
α9α10KO	F	4	Click	22.5	8.660 254
α9α10KO	M	5	4	6	18.165 9
α9α10KO	M	5	8	24	8.944 272
α9α10KO	M	5	12	35.2	6.906 519
α9α10KO	M	5	16	45	7.071 068
α9α10KO	M	5	Click	22	4.472 136

ABR Wave I amplitudes and latencies to 90, 80, and 70 dB SPL clicks were quantified for both genotypes. α9α10KO mice tended to have larger Wave I amplitudes in both quiet and in noise compared to WTs [Fig. 1(C)] and minimal differences in Wave I latency [Fig. 1(D)]. For clicks in quiet, linear mixed-effects, models comparing ABR Wave I amplitudes across genotype, sex, and click level revealed significant main effects of genotype and click level, and significant interactions between genotype/sex, genotype/click level, and genotype/sex/click level (Table I). *Post hoc* analyses revealed a significant difference in quiet Wave I amplitudes between male WTs and male α9α10KOs for 70, 80, and 90 dB SPL clicks (p = 0.0018, 0.0008, and 0.0006, respectively). Linear mixed-effects models comparing ABR Wave I latencies across genotype, sex, and click level revealed a significant main effect of click level and a significant three-way interaction. *Post hoc* analyses identified a significant difference in Wave I latency between female WTs and female α9α10KOs for 90 dB SPL clicks only (p = 0.0475).

For clicks in noise, linear mixed-effects models comparing ABR Wave I amplitudes across genotype, sex, and click level revealed significant main effects of genotype and click level, and significant interactions between genotype/click level and genotype/sex/click level (Table I). *Post hoc* analyses revealed a significant difference in masked Wave I amplitudes between male WTs and male α9α10KOs for 80 and 90 dB SPL clicks only (p = 0.0063 and 0.0006, respectively). Linear mixed-effects models comparing ABR Wave I latencies across genotype, sex, and click level revealed a significant main effect of click level, and significant interactions between genotype/sex and genotype/click level. *Post hoc* analyses of ABR Wave I latencies identified a significant genotype difference for males (p = 0.0024) and a significant genotype difference for 70 dB SPL clicks (p = 0.0440). Overall, the ABR Wave I data for clicks in quiet and in noise suggest that α9α10KO mice had more robust responses compared to WTs.

**FIG. 2.**
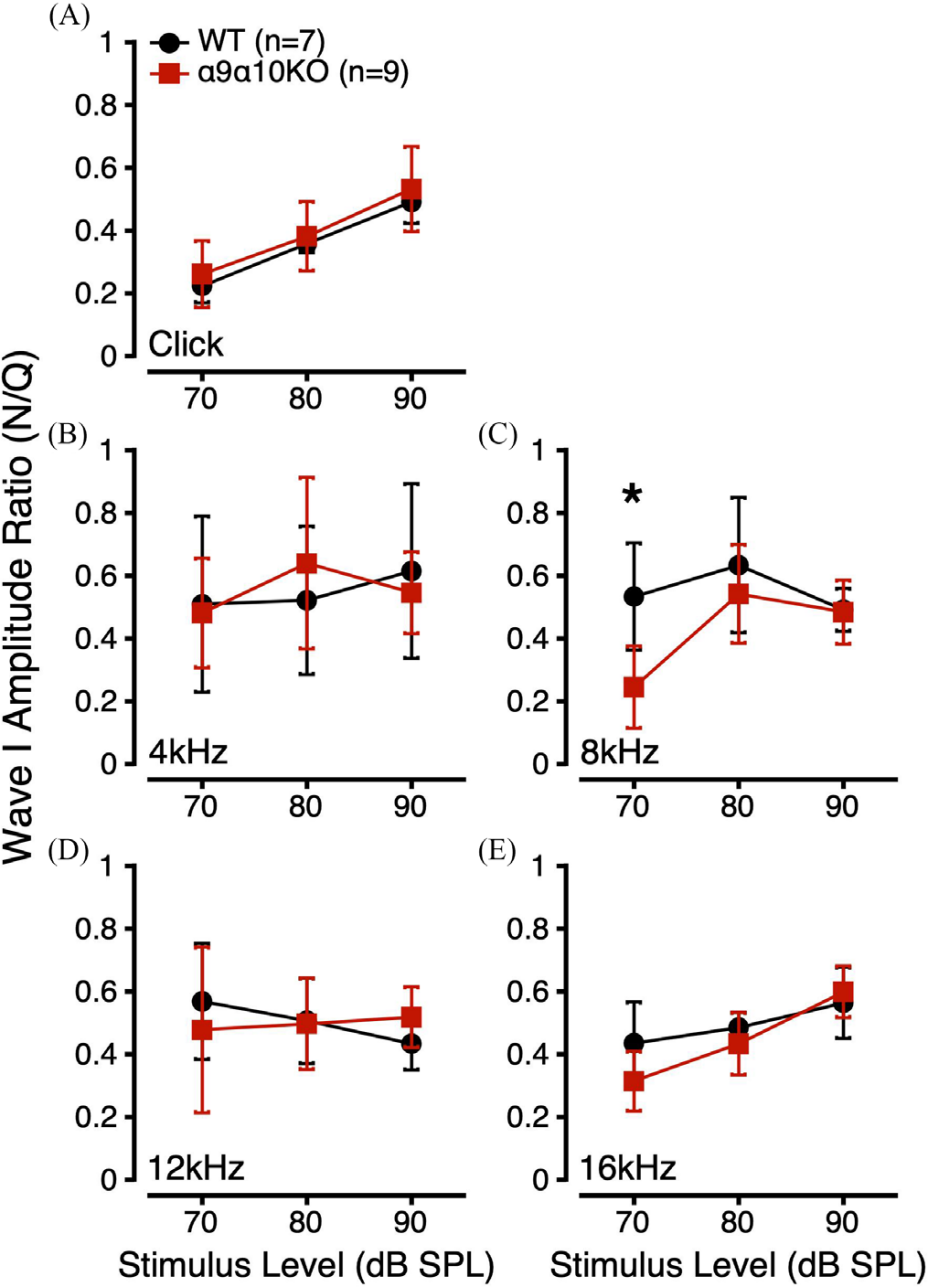
(Color online) ABR Wave I amplitude ratios (*Amplitude_Noise_/Amplitude_Quiet_*) to suprathreshold clicks (A) and tone bursts (B: 4 kHz; C: 8 kHz; D: 12 kHz; E: 16 kHz) for α9α10KO (squares) and WT mice (circles). *indicates a statistically significant difference across genotypes (p-value <0.05).

To further evaluate the effects of masking noise on suprathreshold α9α10KO ABRs, we derived a ratio comparing Wave I amplitude in quiet and in noise for a given stimulus, level, and subject (*Wave I Amplitude Ratio* = *Amplitude_Noise_*/*Amplitude_Quiet_*). The addition of masking noise is expected to reduce Wave I amplitudes, so amplitude ratio values should be less than 1. Wave I amplitude ratios were similar for WT and α9α10KO mice across most stimulus conditions (Fig. 2). Since our masking noise had a constant intensity, the masking noise generally had a greater effect on Wave I amplitudes to lower intensity stimuli [e.g., for the click, Fig. 2(A)].

Linear mixed-effects models were used to compare Wave I amplitude ratios for each stimulus across genotype, stimulus level, and sex. For Wave I amplitude ratios to clicks, this analysis revealed a significant main effect of click level and no significant interactions (Table I). *Post hoc* analyses indicated significant differences in Wave I amplitude ratios for 70, 80, and 90 dB SPL clicks (p < 0.0001 for all). For 8 kHz tone bursts, there were significant main effects of genotype, sex, and stimulus level, and a significant interaction between genotype/stimulus level. Interestingly, *post hoc* analyses revealed a significant genotype difference at 70 dB SPL [p = 0.0008; Fig. 2(C)]. For 16 kHz tone bursts, there was a significant main effect of stimulus level and a significant interaction between genotype/stimulus level. *Post hoc* analyses revealed no significant genotype differences at any stimulus level, although there was a trend toward a significant genotype difference at 70 dB SPL (p = 0.0857). LMEs for 4 and 12 kHz tone burst Wave I amplitude ratios revealed no significant main effects or interactions. Overall, the Wave I amplitude ratio data suggested that suprathreshold α9α10KO ABRs were more susceptible to masking noise at 8 kHz than WTs.

### B. ASRs in quiet and in noise

**FIG. 3.**
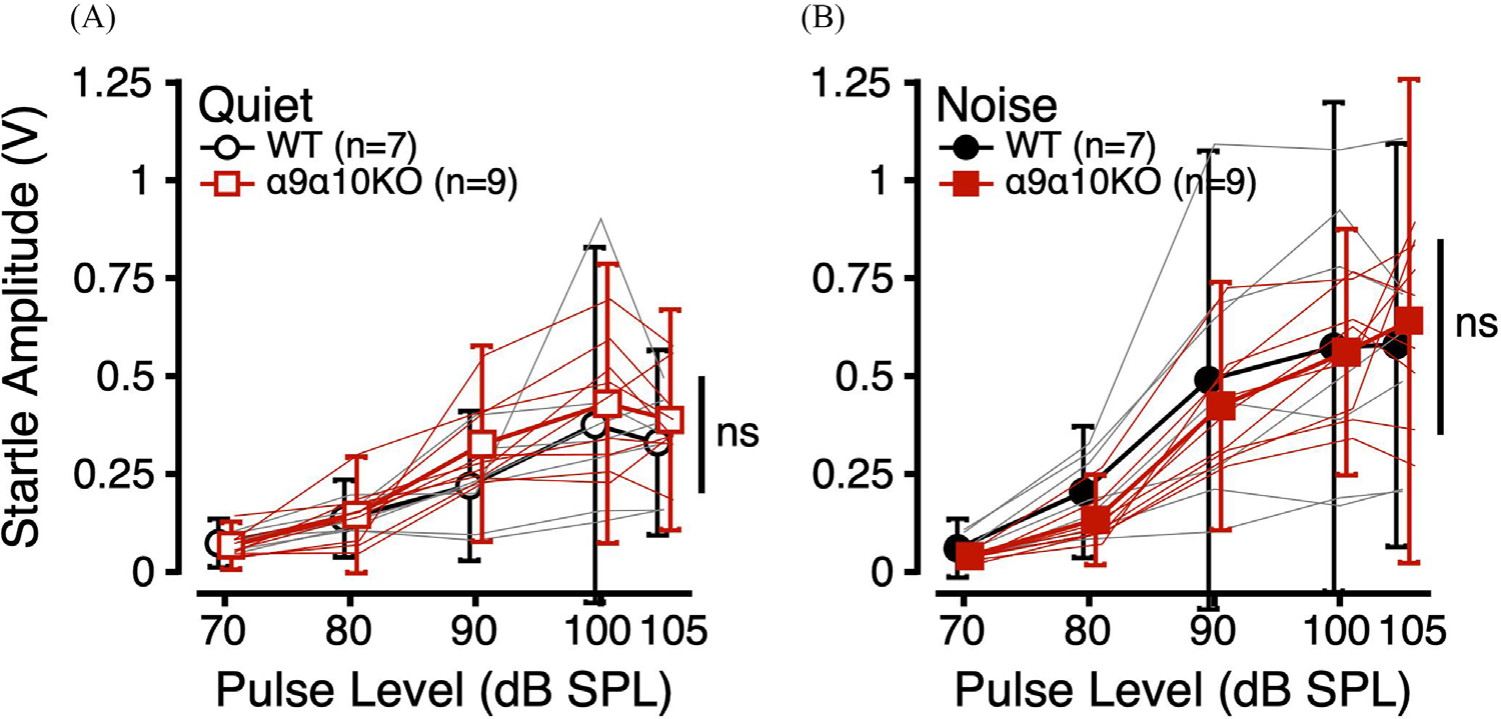
(Color online) ASR amplitudes (V) as a function of pulse level (dB SPL) in quiet (open; A) and in noise (filled; B) for α9α10KO (squares) and WT mice (circles). Thin lines indicate individual subject data.

ASRs were measured to noise bursts of varying intensity in quiet and in 60 dB SPL masking noise. Figure 3 shows mean ASR amplitudes for each genotype as a function of pulse level in quiet (A) and noise (B). Individual data (thin lines) are shown to illustrate the extensive variability in ASR amplitudes across individual animals of both genotypes, as is common for mice. For clarity, only the mean and standard deviation are shown for subsequent figures. As expected, ASR amplitude increased with pulse level across masking conditions and genotypes. ASR amplitudes were similar for WT and α9α10KO mice in both quiet and in noise. For both genotypes, the addition of masking noise increased ASR amplitudes for high-intensity pulses, consistent with previous reports (Carlson and Willott, 2001; Ison, 2001; Kim *et al.*, 2022; McGuire *et al.*, 2015).

Linear mixed-effects models comparing ASR amplitudes in quiet across genotype, sex, and pulse level revealed a significant main effect of pulse level (Table I). *Post hoc* analyses revealed significant differences in ASR amplitude between pulse levels of 70/90, 70/100, 70/105, 80/90, 80/100, 80/105, 90/100 (p < 0.0001 for all), and 90/105 (p = 0.239). Similarly, linear mixed-effects models comparing ASR amplitudes in noise revealed a significant main effect of pulse level, with *post hoc* analyses showing significant differences in ASR amplitude between pulse levels of 70/80 (p = 0.0433), 70/90, 70/100, 70/105, 70/105, 80/90, 80/100, 80/105 (p < 0.0001 for all), 90/100 (p = 0.0464), and 90/105 (p = 0.0100). These results suggest that ASR amplitudes peaked at 100 and 105 dB SPL and that there were no differences in ASR amplitudes between WT and α9α10KO mice in quiet or in noise.

### C. PPI of the ASR: FD sensitivity

**FIG. 4.**
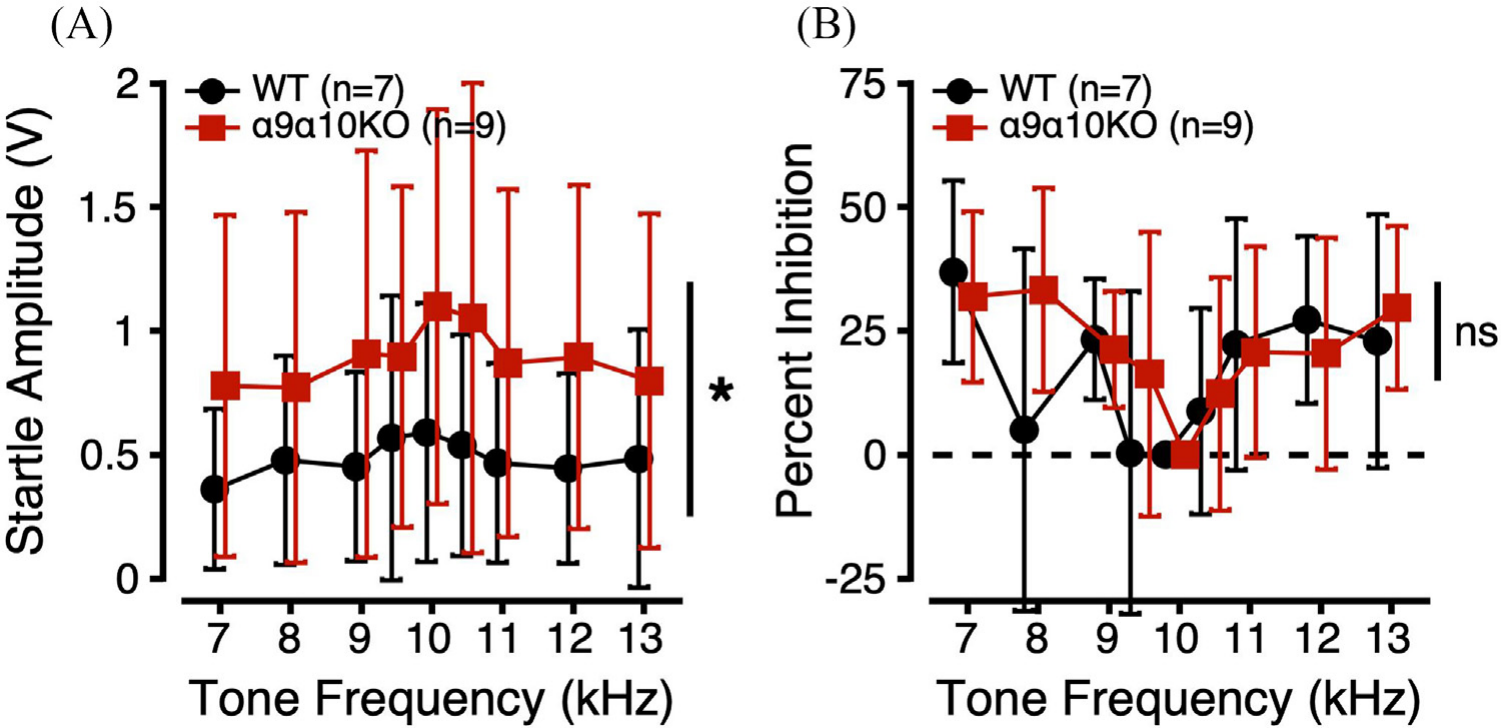
(Color online) FD sensitivity estimated from PPI of the ASR for α9α10KO (squares) and WT mice (circles). (A) ASR amplitudes (V) as a function of prepulse cue frequency (kHz). (B) Percent inhibition of the ASR as a function of prepulse cue frequency. *indicates a statistically significant difference across genotypes (p-value <0.05).

PPI of the ASR was used to probe sensitivity to FDs. In this task, startle-inducing noise bursts presented in a 70 dB SPL 10 kHz tone background were preceded by a change in background tone frequency. If the mouse is able to perceive this prepulse cue, they should inhibit their startle to the oncoming noise burst. Startle amplitudes are plotted as a function of prepulse frequency in Fig. 4(A). Startle amplitudes were variable across and within genotypes, but generally decreased as the prepulse frequency deviated farther from 10 kHz. α9α10KO mice had larger startle amplitudes across all FD conditions compared to WTs. Linear mixed-effects models comparing startle amplitudes as a function of genotype, sex, and prepulse frequency revealed significant main effects of genotype and frequency. *Post hoc* analyses revealed a trend toward a significant difference in startle amplitude across genotype (p = 0.0538) and significant differences in startle amplitude between prepulse frequencies of 7/10 (p = 0.0001), 7/10.5 (p = 0.0049), 8/10 (p = 0.0046), and 10/13 (p = 0.0175). These results suggest that α9α10KO mice may be hyperreactive to certain acoustic startle conditions compared to WTs.

PPI of the ASR was calculated according to: *Percent Inhibition* =  (*Startle_no_change_* – *Startle_change_*/*Startle_no_change_*) × 100. When an animal inhibits its ASR, the PPI value should be greater than 0. In Fig. 4(B), PPI is plotted as a function of prepulse frequency. Despite the α9α10KO mice having larger raw ASR values in the FD task, they showed similar PPI to the WTs [Fig. 4(B)]. Linear mixed-effects models comparing PPI as a function of genotype, sex, and prepulse frequency revealed a significant main effect of frequency (Table I). *Post hoc* analyses revealed significant differences in PPI between prepulse frequencies of 7/9.5 (p = 0.0022), 7/10 (p < 0.0001), 7/10.5 (p = 0.0076), 9/10 (p = 0.0174), 10/11 (p = 0.0256), 10/12 (p = 0.0076), and 10/13 kHz (p = 0.0019). These results suggest that WT and α9α10KO mice have similar FD sensitivity.

### D. PPI of the ASR: ID sensitivity

**FIG. 5.**
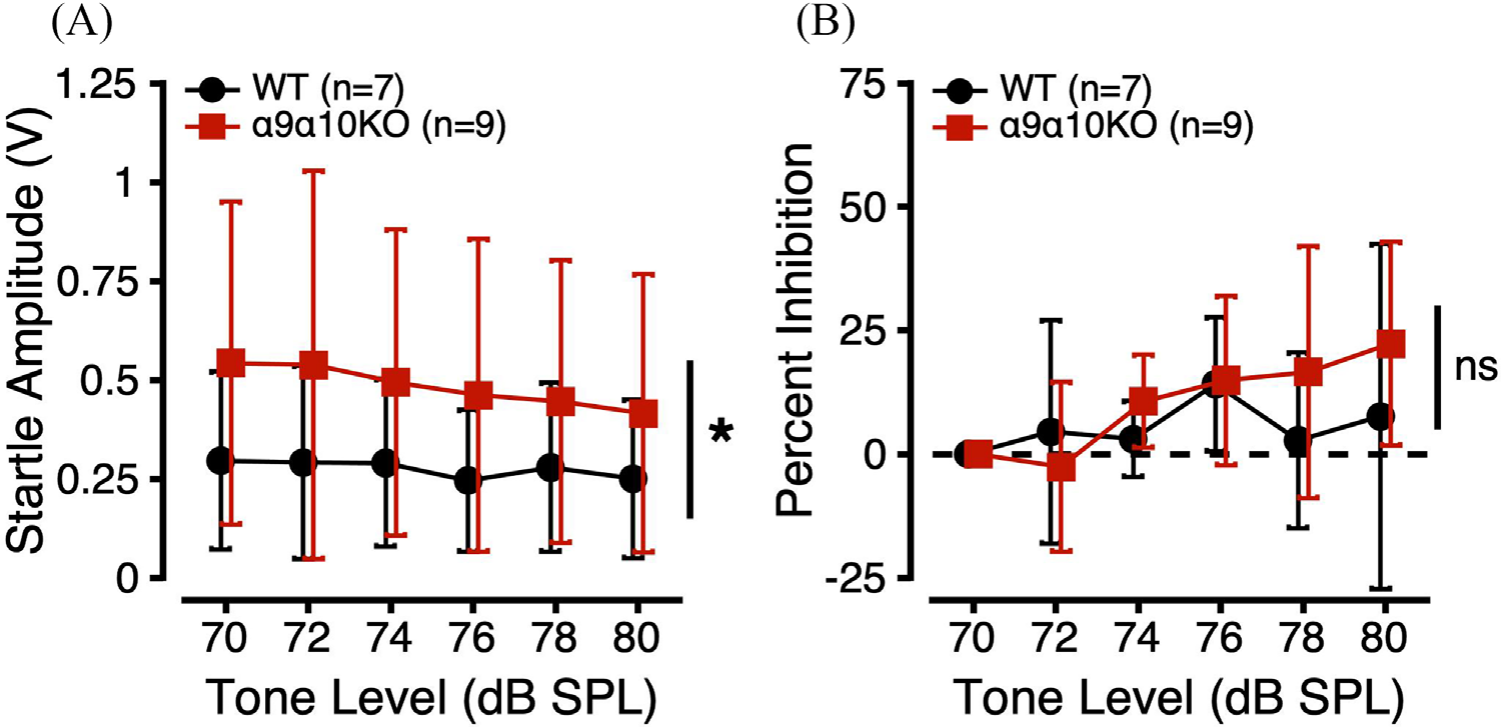
(Color online) ID sensitivity estimated from PPI of the ASR for α9α10KO (squares) and WT mice (circles). (A) ASR amplitudes (V) as a function of prepulse cue level (dB SPL). (B) Percent inhibition of the ASR as a function of prepulse cue level. *indicates a statistically significant difference across genotypes (p-value <0.05).

PPI of the ASR was also used to probe sensitivity to IDs. In this task, startle-inducing noise bursts presented in a 70 dB SPL 10 kHz tone background were preceded by a change in background tone level. Startle amplitudes are plotted as a function of prepulse level in Fig. 5(A). As with the FD task, startle amplitudes in the ID task were variable across and within genotypes, but generally decreased as the prepulse level deviated farther from 70 dB SPL. α9α10KO mice had larger startle amplitudes than WT mice across all ID conditions. Linear mixed-effects models comparing startle amplitudes as a function of genotype, sex, and prepulse level revealed significant main effects of genotype, sex, and level, but no significant interactions (Table I). *Post hoc* analyses revealed significant differences in startle amplitude across genotype (p = 0.0292) and between prepulse levels of 70/80 (p = 0.0347) and 72/80 (p = 0.0498). Consistent with the FD testing, these results again suggest that α9α10KO mice may have exaggerated startle responses to some acoustic stimuli compared to WTs.

In Fig. 5(B), PPI is plotted as a function of prepulse level. Despite the α9α10KO mice having larger raw ASR values in the ID task, they showed similar PPI to the WTs [Fig. 5(B)]. Linear mixed-effects models comparing PPI as a function of genotype, sex, and prepulse level revealed a significant main effect of prepulse level. However, no *post hoc* comparisons were significant. These results suggest that WT and α9α10KO mice have similar ID sensitivity.

## IV. DISCUSSION

We further characterized auditory function in the α9α10KO mouse model with deficient MOC inhibition of outer hair cells by probing hearing-in-noise and suprathreshold processing abilities. We combined physiological (ABRs in quiet and in noise) and reflex-based behavioral measures (ASRs in quiet and in noise, PPI measures of frequency and ID sensitivity) that are commonly used in hearing studies and have been examined in other MOC mutants. Consistent with previous findings in α9KOs, α9α10KO mice showed normal ABR thresholds in quiet, normal ID sensitivity, and hyperreactivity to sounds under some ASR and PPI stimulus conditions (Clause *et al.*, 2017; Lauer, 2017; Lauer and May, 2011; Vetter *et al.*, 1999). However, in contrast to predictions about impaired MOC function, α9α10KO mice showed normal ABR thresholds in noise, enhanced ABR amplitudes in quiet and in noise, and grossly normal FD sensitivity compared to WTs. We infer that α9α10KO mice may develop compensatory mechanisms that support auditory function in the absence of normal MOC function, as has been suggested for α9KO mice (May *et al.*, 2002). This study contributes to the growing, but conflicting, literature on the role of the MOC system in hearing in noise (Lauer *et al.*, 2022).

### A. Physiological responses to signals in quiet and noise

We used ABRs in quiet and in noise as physiological estimates of hearing sensitivity and hearing-in-noise function. ABR thresholds in quiet and in noise suggested comparable hearing sensitivity for α9α10KOs and WTs, consistent with previous reports in α9KOs (Lauer, 2017; Lauer and May, 2011; Morley *et al.*, 2017). ABR wave amplitudes and latencies were also examined to evaluate whether morphological differences were apparent across genotypes. Wave morphology was highly variable, especially for central waves, so we focused our analyses on ABR Wave I only. The larger Wave I amplitudes observed for α9α10KO mice than WTs may be due to the lack of efferent suppression of transient tone stimuli or developmental compensatory processes in the α9α10KOs.

Conflicting results were observed across ABR metrics of hearing-in-noise function. Some comparisons indicated that α9α10KOs were more resistant to masking effects than WTs (dB masking), whereas other measures suggested that α9α10KOs were more susceptible to masking noise than WTs (Wave I amplitude ratio), consistent with weaker MOC-mediated noise suppression. Although these metrics were statistically different across genotypes, group sizes were small and the differences were specific to interactions among stimulus frequency, level, and/or sex that were not consistent across the two ABR metrics (Table I). Therefore, it is difficult to identify a biologically relevant interpretation for these seemingly spurious findings. Since C57BL/6J mice lose their MOC responses as early as 8 weeks, despite intact responses at 6 weeks (Zhu *et al.*, 2007), it is possible that genotype differences in the ABR would be more apparent or consistent in slightly younger animals (Morley *et al.*, 2017).

### B. Behavioral responses to signals in quiet and noise

We measured behavioral responses to sounds in quiet and in noise using ASR and PPI techniques to probe hearing-in-noise function. We utilized these measures to avoid repeated exposure to sounds and practice effects that occur with traditional rodent psychoacoustic tasks out of a concern for potential behavioral compensation (Lauer *et al.*, 2017; May *et al.*, 2002). Previous experiments in α9KO mice on a range of background strains have shown abnormally large prepulse facilitation in response to short gaps in noise (Lauer and May, 2011), reduced PPI in quiet but not noise (Allen and Luebke, 2017), and reduced PPI to changes in frequency, but not intensity (Clause *et al.*, 2017). In contrast, we found no differences in PPI to frequency or intensity changes in α9α10KO mice. Although PPI methodology is limited in its ability to assess acuity (Lauer *et al.*, 2017), we were surprised to find no gross differences here. One possible contribution to these discrepancies is variations in the onset of age-related hearing loss and MOC decline across mice from different background strains (e.g., CBA/CaJ vs C57BL/6J) (Ohlemiller, 2019; Zhu *et al.*, 2007). Differing phenotypes across studies of MOC mutants point to the need for experiments to clarify how MOC function may interact with genes that vary across background strains and breeding strategies.

However, α9α10KO mice showed larger than normal ASR amplitudes in the presence of constant background tones, indicating increased salience of the startle-eliciting stimuli under these conditions. Animal weights were similar across genotypes, as were the ASR amplitudes in quiet and broadband noise backgrounds. Thus, we cannot attribute the effect to differences in mass reactivity on the ASR apparatus. Although α9 and α10 subunits are expressed in other tissues, including vestibular organs (Poppi *et al.*, 2020), there is no verified functional expression in the brain. However, central ASR circuitry may have developed abnormally in the α9α10KO mice, regardless of receptor expression. The hyperreactivity to abrupt, loud sounds could indicate a form of loudness hyperacusis experienced in the presence of background sounds with starkly different spectral content. Prior studies have shown ASR potentiation in the presence of background sounds (Basavaraj and Yan, 2012; Carlson and Willott, 2001; Gerrard and Ison, 1990; Hoffman and Fleshler, 1963). The background tone might normally trigger efferent suppression of the response to the broadband startle-eliciting stimulus, an effect which is absent in the α9α10KO mice. Interestingly, hyperreactivity was not apparent for ASRs in the presence of constant background noise, despite similar levels for the noise and tone (60 and 65 dB SPL, respectively). This discrepancy may be due to the energy of the noise being dispersed across a broad frequency range, rather than concentrated at a single frequency for the tone, similar to reports of hyperacusis for specific sounds (Tyler *et al.*, 2014).

### C. Sex differences in MOC mutant mice

Sex differences in auditory system structure and function are poorly understood, partially due to sex bias and omission in basic science research (Villavisanis *et al.*, 2020). In the present study, mice of both sexes were included, but modest group sizes limit our ability to draw clear conclusions about these differences. Sex effects may have contributed to the physiological differences between α9α10KOs and WTs (Dondzillo *et al.*, 2021), as male mice exhibited a greater number of differences across genotypes than females. However, the sporadic statistically significant findings across our full test battery (Table I) did not support a systematic sex difference in MOC dysfunction. Similar spurious findings have been reported in physiological and behavioral studies of α9KO mice (Lauer, 2017; Lauer and May, 2011), with little consistency across stimulus frequency or sex. While these results should not be ignored, they should also not be overemphasized in the absence of specific hypotheses. Research investigating sex- and frequency-specific differences in olivocochlear function and hearing-in-noise abilities should be conducted in large cohorts of normal hearing mouse models to explore the origins of the differences reported here.

### D. Potential compensatory mechanisms in MOC mutant mice

Overall, α9α10KO mice exhibited variable responses to signals in noise, so it is unclear whether they are more or less susceptible to masking effects. The larger ABR Wave I amplitudes and larger ASR amplitudes suggest compensation and/or hyperreactivity in α9α10KO mice. One potential mechanism for preserving hearing function in α9α10KO mice is activity-dependent plasticity in the lateral olivocochlear efferents (Frank *et al.*, 2023; Niu and Canlon, 2002; Wu *et al.*, 2020). A previous study reported qualitatively normal ChAT staining of lateral olivocochlear synapses in α9α10KO mice (Morley *et al.*, 2017; not quantified), but changes in other neurotransmitter systems have not been investigated. It is feasible that, amidst diminished MOC feedback, neurotransmitter expression in the lateral efferent neurons adjusts to compensate for the lack of medial efferent-mediated cochlear gain control. This could result in preserved ABR thresholds in quiet and noise via direct modulation of auditory nerve activity. The role of the lateral olivocochlear neurons on more complex aspects of hearing behavior is entirely unknown, since specific manipulations of this pathway have not been applied in psychoacoustic experiments (but see Allen and Luebke, 2017, for some potential lateral efferent-mediated effects involving calcitonin gene-related peptide).

Other compensation mechanisms contributing to α9α10KO phenotypes may include plasticity of the central auditory pathway. The absence of MOC gain control in α9α10KO mice is like having a chronic noise exposure, since background noises will not be attenuated. This also results in an increase in afferent activity. Enhanced auditory input is known to broadly and significantly impact physiology in the brainstem and auditory cortex (Ngodup *et al.*, 2015; Occelli *et al.*, 2022; Oliver *et al.*, 2011; Willott *et al.*, 2005; Zhang *et al.*, 2001). Specific contributions from peripheral and central compensatory mechanisms to hearing-in-noise phenotypes in MOC mutants are unknown and should be explored in future studies.

## V. CONCLUSION

The persistent difficulty in identifying clear and consistent hearing deficits in genetically mutated mice with impaired MOC activity, as well as in behaviorally trained animals with surgical lesions of the MOC system (e.g., Dewson, 1967; Igarashi *et al.*, 1972; May and McQuone, 1995; Trahiotis and Elliott, 1970), underscores the need for more specific, controlled, acute manipulations to elucidate the effects on hearing. It is perhaps unsurprising that different functional outcomes have been reported in α9KO, α10KO, and α9α10KO mice, given differences in genetic background, development, age at testing, environmental housing conditions, ambient sound exposure histories, and test measurements across studies. Future studies should make direct phenotype comparisons across single and double KO mutants to better understand which effects are due to the absence of α9 vs α10 receptor subunits while controlling for extraneous factors. Additionally, genetically engineered genotypes (e.g., α9α10KOs mutants) inherently develop with diminished or enhanced MOC activity. To overcome this limitation, future experiments could make use of virally introduced genetic gain-of-function mutations (Zhang *et al.*, 2023), inducible gene KOs, DREADDS, or optogenetic stimulation or silencing of MOC neurons in adult animals. Finally, potential compensatory mechanisms, such as lateral olivocochlear plasticity, should be further investigated, as these mechanisms have important implications for listeners with degenerating MOC systems due to age, noise, and other damaging circumstances (Lauer, 2017; Lauer *et al.*, 2022; Vicencio-Jimenez *et al.*, 2021).

## Data Availability

The data that support the findings of this study will be publicly available on the Johns Hopkins University data repository following publication of the manuscript. Interested parties may contact the authors for additional data inquiries.
